# Chronic Treatment with Squid Phosphatidylserine Activates Glucose Uptake and Ameliorates TMT-Induced Cognitive Deficit in Rats via Activation of Cholinergic Systems

**DOI:** 10.1155/2012/601018

**Published:** 2012-05-22

**Authors:** Hyun-Jung Park, Seung Youn Lee, Hyun Soo Shim, Jin Su Kim, Kyung Soo Kim, Insop Shim

**Affiliations:** ^1^Acupuncture and Meridian Science Research Center, Kyung Hee University, Seoul 130-701, Republic of Korea; ^2^Department of Family Medicine, The Catholic University of Korea, Seoul 137-701, Republic of Korea; ^3^Molecular Imaging Research Center, Korea Institute of Radiological & Medical Sciences, University of Science & Technology, Republic of Korea

## Abstract

The present study examined the effects of squid phosphatidylserine (Squid-PS) on the learning and memory function and the neural activity in rats with TMT-induced memory deficits. The rats were administered saline or squid derived Squid-PS (Squid-PS 50 mg kg^−1^, *p.o.*) daily for 21 days. The cognitive improving efficacy of Squid-PS on the amnesic rats, which was induced by TMT, was investigated by assessing the passive avoidance task and by performing choline acetyltransferase (ChAT) and acetylcholinesterase (AchE) immunohistochemistry. 18F-Fluorodeoxyglucose and performed a positron emission tomography (PET) scan was also performed. In the passive avoidance test, the control group which were injected with TMT showed a markedly lower latency time than the non-treated normal group (*P* < 0.05). However, treatment of Squid-PS significantly recovered the impairment of memory compared to the control group (*P* < 0.05). Consistent with the behavioral data, Squid-PS significantly alleviated the loss of ChAT immunoreactive neurons in the hippocampal CA3 compared to that of the control group (*P* < 0.01). Also, Squid-PS significantly increased the AchE positive neurons in the hippocampal CA1 and CA3. In the PET analysis, Squid-PS treatment increased the glucose uptake more than twofold in the frontal lobe and the hippocampus (*P* < 0.05, resp.). These results suggest that Squid-PS may be useful for improving the cognitive function via regulation of cholinergic enzyme activity and neural activity.

## 1. Introduction

Trimethyltin (TMT) is an organotin compound with potent neurotoxicant effects. This substance is regarded as being particularly useful for studying the response to injury on account of the distinct pattern of degeneration it causes in rodent brain. In particular, the rat hippocampus constitutes the most suitable model for TMT-induced brain injury [1–4]. The molecular basis for the selective vulnerability of specific neuronal populations to neuronal insults has been a key focus in the fields of neurology and neuropathology [5]. TMT-induced neurodegeneration is characterized by massive neuronal death that is mainly localized in the limbic system and especially in the hippocampus, and this is accompanied by reactive gliosis, epilepsy, and marked neurobehavioral alterations, and so this is considered a useful model of neurodegeneration and selective neuronal death [5–11]. Also, in rats, TMT induces the degeneration of pyramidal neurons in the hippocampus and the cortical areas (pyriform cortex, entorhinal cortex, and subiculum) connected to the hippocampus, but there is also neuronal loss in the association areas [6, 12, 13]. Furthermore, behavioral studies have shown increased locomotor activity, disruption in self-grooming, and learning deficits in TMT-intoxicated rats [1, 5, 14–21]. TMT intoxication impairs the performance of learning acquisition of water maze and Biel maze (water avoidance) tasks as well as the performance of Hebb-Williams maze and radial arm maze tasks. In addition, TMT intoxication produces deficits in passive avoidance retention, but not in the acquisition of the passive avoidance response [2–4, 10, 21, 22]. Furthermore, deficits in the acquisition of active avoidance at the beginning of training have been reported [16]. Moreover, TMT has been shown to produce effects on operant behavior since TMT-intoxicated rats had higher rates of lever pressing under a fixed-ratio schedule of food presentation [20], and TMT impaired the performance of differential reinforcement at low response rates in an operant schedule [23]. These anatomical and behavioral findings have made TMT-intoxicated rats an attractive model for degenerative diseases such as AD, which is the most common cause of dementia [16].

Phosphatidylserine (PS), a phospholipid nutrient, is active in cell membranes and is the major acidic phospholipid component in the membranes of the brain. Membranes are the working surfaces of every cell, carrying out the essential functions of cellular communication and hormonal signal transduction [24, 25]. Nerve cells, in particular, depend on healthy membrane function for normal neurotransmitter metabolism and nerve signal transmission [26]. Also, PS assists in maintaining adequate glucose utilization in the brain. Glucose is the preferred energy substrate for nerve cells which, unlike other cells, are unable to use fatty acids or proteins for energy production. Brain glucose utilization, an indicator of brain activity, often declines during aging [27].

The present study was undertaken to evaluate the neuroprotective effect of Squid-PS on the TMT-induced memory deficit in rats and to elucidate the mechanism underlying these protective effects in rats. Rats were tested on a passive avoidance test for learning and memory. The analyzed parameters included the expression of cholinergic neurons and neural activity in the hippocampus.

## 2. Materials and Methods

### 2.1. Animals and the Experimental Design

Male Sprague-Dawley rats weighting 250–280 g (8 weeks old) each were purchased from Samtaco Animal Corp. (Kyungki-do, Korea). The animals were housed in individual cages under light-controlled conditions (12/12-hour light/dark cycle) and at 23°C room temperature. Food and water were made available ad libitum. All the experiments were approved by the Kyung Hee University institutional animal care and use committee. Also, this experimental protocol was approved by an Institutional Review Committee for the Use of Human or Animal Subjects or that procedures are in compliance with at least the Declaration of Helsinki for human subjects, the National Institutes of Health Guide for Care and Use of Laboratory Animals (Publication no. 85-23, revised 1985), the UK Animals Scientific Procedures Act 1986, or the European Communities Council Directive of 24 November 1986 (86/609/EEC). The rats were allowed at least 1 week to adapt to their environment before the experiments.

The rats were injected intraperitoneally (i.p.) with TMT (8.0 mg/kg, body weight) dissolved in 0.9% saline, and then they were returned to their home cages.

The rats were randomly assigned to three groups of six individuals each as follows: nontreated, naïve normal group (normal); saline-treated group (control); 50 mg kg^−1^ Squid-PS-treated group (Squid-PS 50) used in this study, which were manufactured and kindly provided by Doosan Co. Glonet BU (Youngin, Korea). The rats were orally administrated with PS, daily for 21 days.

### 2.2. Passive Avoidance Task (PAT)

A passive avoidance task was performed after 21 days of the administration of Squid-PS. Rats were trained in a step-through inhibitory avoidance task. On the training trial, each rat was placed on a lighted platform outside a hole leading to a dark compartment. When the rat stepped into the dark compartment, a constant current foot shock (5 V, 0.5 mA, 10 seconds) was delivered twice. For the retention test, at 24 hours (day1), and at 2 and 3 days later, each rat was again placed on the platform, and the latency to step through was recorded.

### 2.3. Immunohistochemistry

Briefly, the rats were anesthetized (sodium pentobarbital, 100 mg/kg, IP) and then perfused transcardially with heparinized phosphate-buffered saline (PBS; pH 7.4) for 30 min followed by 4% paraformaldehyde in 0.1 M phosphate buffer (pH 7.4) for 10–15 min. The brains were postfixed in the same fixative overnight, cryoprotected in 30% sucrose solution in PBS, embedded and serially sectioned on a cryostat (Leica, Germany) at 30 *μ*m thickness in the coronal plane, and were collected in PBS. The primary antibodies against the following specific antigen were used: cholinacetyl transferase (sheep polyclonal ChAT, concentration 1 : 2000; Cambridge Research Biochemicals, Cleveland, UK) and acetylcholinesterase (rabbit polyclonal AchE, concentration 1 : 1000; Cambridge Research Biochemicals, Cleveland, UK). The primary antibody was prepared and diluted in 0.3% PBST, 2% blocking serum, and 0.001% kehole limpet hemocyanin (Sigma, USA). The sections were incubated in the primary antiserum for 72 h at 4°C. Following rinsing in PBST, sections were incubated for 2 hr at room temperature in biotinylated rabbit anti-sheep or antirabbit serum (Vector Laboratories, Burlingame, CA, USA) diluted 200X in PBST containing 2% normal rabbit serum. Following a further rinsing in PBS, the tissue was developed using diaminobenzidine (Sigma, USA) as the chromogen. The images were captured using a DP2-BSW imaging system (Olympus, CA, USA), and they were processed using Adobe Photoshop. For measuring the cells that were positive for ChAT and AchE, the grid was placed on CA1 and CA3 in the hippocampus area according to the method of Paxinos et al. [28]. The number of cells was counted at 100x magnification using a microscope rectangle grid that measured 200 × 200 mm. The cells were counted in three sections per rat within the hippocampal CA1 and CA3 areas. The brain sections were visually inspected at 3 different anteroposterior levels extending from −2.12 to −6.04 mm [28].

### 2.4. [F-18]FDG Micro-PET Scan

All the rats were deprived of food for 12–15 h before the experiments to enhance the [F-18] FDG uptake in the brain [20]. Each animal was placed on a heating pad in a cage and warmed for at least 30 min before the [F-18] FDG injection. The temperature of the cages was kept at 30°C throughout the uptake period in accordance with an optimized [F-18] FDG uptake protocol [20]. [F-18] FDG (500 *μ*Ci/100 g body weight) was injected through a tail vein, and the rats were anesthetized with 2% isoflurane in 100% oxygen (Forane solution; ChoongWae Pharma). For the PET imaging, a Siemens Inveon PET scanner (Siemens Medical Solutions, USA) was used throughout the study. The transverse resolution that was used was <1.8 mm at the center [20, 29].

The transmission PET data was acquired for 15 min using a Co-57 point source with an energy window of 120~125 keV. One mCi of [F-18] FDG was injected. After allowing for 30 min of tracer uptake time, 30 min of emission PET data was acquired within an energy window of 350~650 keV. The emission list-mode PET data was sorted into 3D sinograms and reconstructed using 3 DRP methods. The pixel size of the reconstructed image was 0.15 × 0.15 × 0.79 mm^3^. Attenuation and scatter corrections were performed for all the datasets. Automated regions of interest (ROI) were used to sample cerebral metabolic rate for glucose from the spatially normalized PET within specific AD-related brain regions (hippocampus, prefrontal cortex).

### 2.5. Voxel-Based Statistical Analysis

Voxel-based statistical analysis was performed to compare the cerebral glucose metabolism of the Squid-PS and control datasets. The procedure used for SPM analysis of the animal PET data was as previously described in our previous study [29]. Briefly, for efficient spatial normalization, only the brain region was extracted. A study-specific template was then constructed using all the datasets. The PET data was spatially normalized onto a rat brain template and smoothed using a 3 mm Gaussian kernel. Count normalization was performed. A voxel-wised *t*-test between the Squid-PS and normal datasets was performed using the Statistical Parametric Mapping 5 program (*P* < 0.05, *K* > 50).

### 2.6. Statistical Analysis

Statistical comparisons were done for the behavioral and histochemical studies using one-way or two-way ANOVA and repeated measures of ANOVA, respectively, and Tukey's *post hoc* test was done. All of the results are presented as means ± SEM, and SPSS 15.0 for Windows was used for analysis of the statistics. The significance level was set at *P* < 0.05. 

## 3. Results

### 3.1. Effect of Squid-PS on the Performance of the Passive Avoidance Task

**Figure 1:**
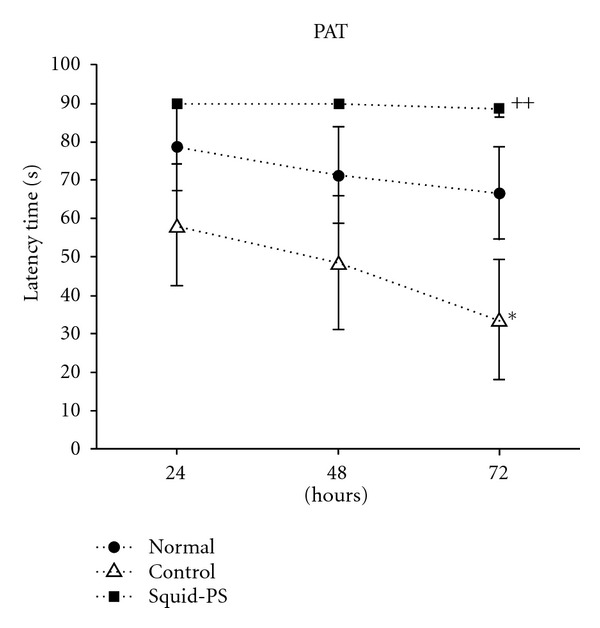
The effect of Squid-PS on passive avoidance task. Data represent means ± SEM of the latency in the passive avoidance task, on learning and memory performance. Rats were exposed to the acquisition trial (DAY1), and retention tests were performed on the following two days (DAY2-3). Statistics: one-way ANOVA test, followed by LSD test. *P < 0.05 as compared with the corresponding data of normal group. ^++^ *P* < 0.01 as compared with the corresponding data of control group.

As shown in Figure 1, the control group showed a memory deficit in the passive avoidance test compared to the normal group (*F*_2,11_ = 2.68, *P* < 0.05). However, the Squid-PS group showed improved memory compared to the control (Day 3, *P* < 0.01).

### 3.2. ChAT Immunoreactive Neurons of the Hippocampus

**Figure 2:**
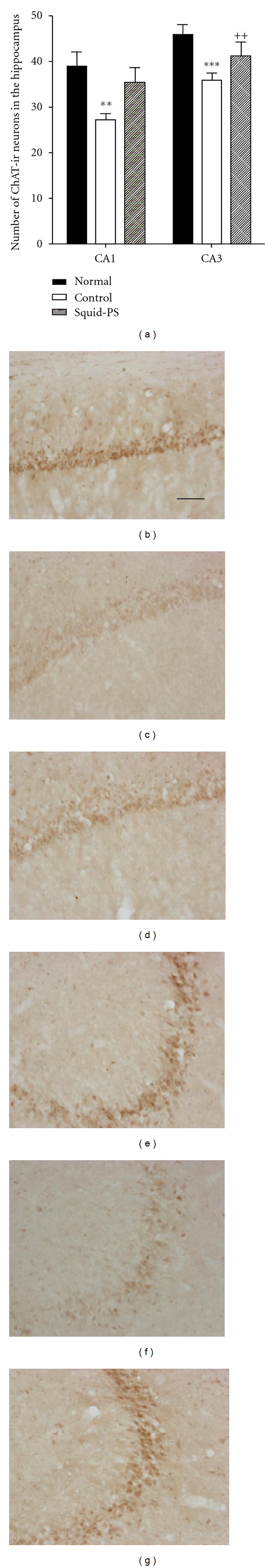
(a) The photograph of ChAT immunoreactivity on hippocampus. (b, e) normal group, (c, f) control group, and (d, g) Squid-PS group ChAT expression on the hippocampus. Each column represents the mean value ± SEM per group. Statistics: one-way ANOVA test, followed by LSD test. **P < 0.01, ***P < 0.001 as compared with the corresponding data of normal group. ^++^ *P* < 0.01 as compared with the corresponding data of control group.

ChAT immunoreactive cells in the different hippocampal subregions are shown in Figure 2. Post-hoc comparisons indicated that the ChAT immunoreactivity in the hippocampal CA1 and CA3 of the control group was significantly reduced compared with that of the normal group (*F*_2,16_ = 5.4, *P* < 0.05; *F*_2,16_ = 15.6, *P* < 0.001). Also, ChAT reactivity in the Squid-PS group was higher than that of the control group, and particularly in CA3 (*P* < 0.01).

### 3.3. AchE Immunoreactive Neurons of the Hippocampus

**Figure 3:**
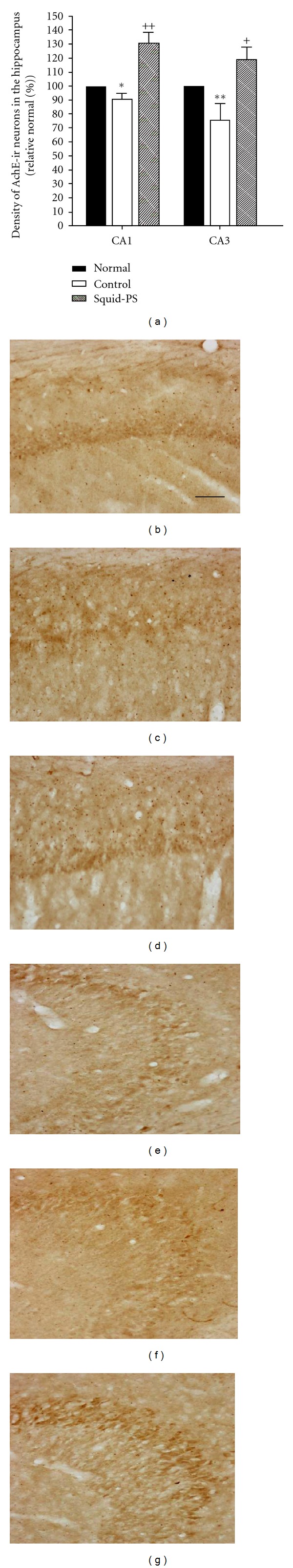
(a) The photograph of AchE immunoreactivity on hippocampus. (b, e) normal group, (c, f) control group, and (d, g) Squid-PS group AchE expression on the hippocampus. Each column represents the mean value ± SEM per group. Statistics: one-way ANOVA test, followed by LSD test. *P < 0.05, **P < 0.01 as compared with the corresponding data of normal group. ^+^ *P* < 0.05, ^++^ *P* < 0.01 as compared with the corresponding data of control group.

AchE immunoreactive cells in the different hippocampal subregions are shown in Figure 3. Post hoc comparisons indicated that the AchE activity in the hippocampus of the control group was significantly lower than that of the normal group (*P* < 0.01). In particular, there were significant differences in both CA1 (*F*_2,13_ = 7.3, *P* < 0.01) and CA3 (*F*_2,13_ = 7.6, *P* < 0.01). However, the AchE reactivity in the Squid-PS group was higher than that of the control group, and particularly in CA1 (*P* < 0.01) and CA3 (*P* < 0.05).

### 3.4. Change in Brain Glucose Metabolism

**Figure 4:**
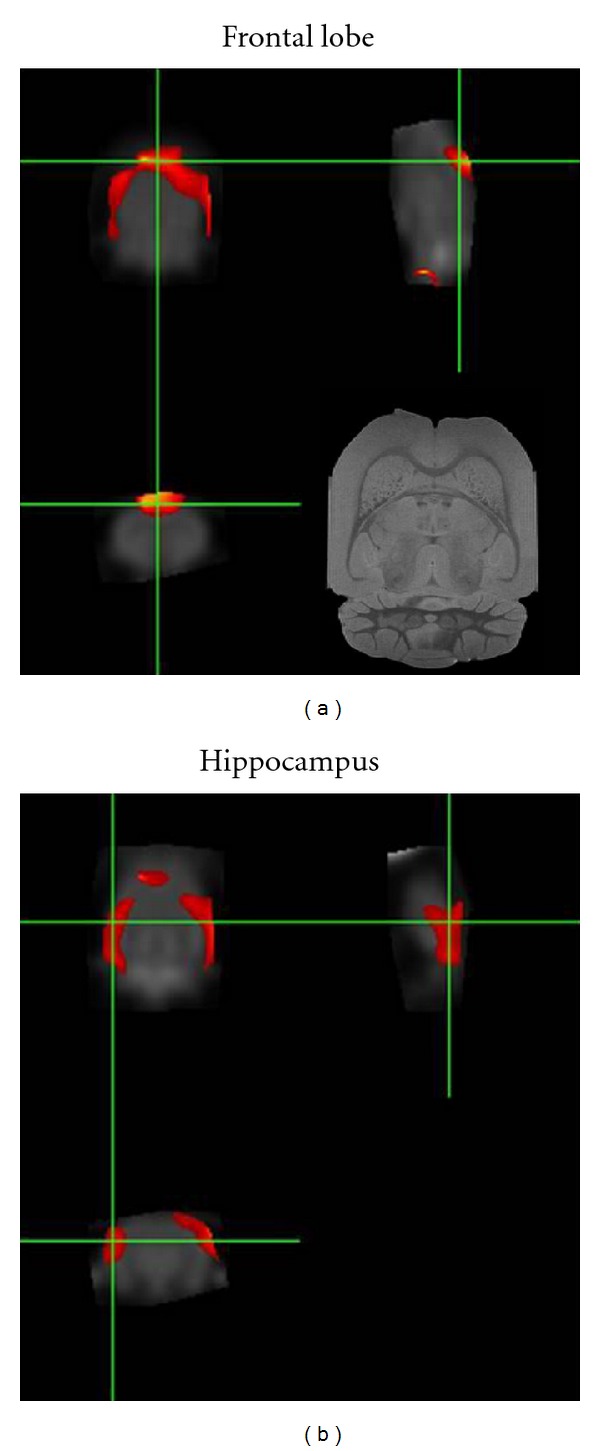
Brain regions where regional FDG uptakes in Squid-PS were significantly higher than in control (hippocampus and frontal lobe).

The analysis of the brain glucose metabolism analysis was shown in Figure 4, and Table 1. The result of voxel-wised shows comparison between the Squid-PS and control datasets. FDG-PET image scans indicated differences in the cerebral metabolic rate of glucose from the hippocampus to prefrontal cortices between the control and Squid-PS group. On the SPM analysis, the glucose metabolism of the Squid-PS datasets was significantly increased in the hippocampus and frontal lobe compared to that of the control (*P* < 0.05).

## 4. Discussion

The present study demonstrated that TMT injections produced severe deficits in the rats' performances in a passive avoidance task along with signs of neurodegeneration, including decreased cholinergic neurons and neural glucose activity in the hippocampus. Repeated treatment with Squid-PS attenuated the TMT-induced learning and memory deficits in the passive avoidance task, and it had a protective effect against the TMT-induced decrease in cholinergic positive neurons. Also, Squid-PS increased the glucose uptake approximately twofold in the frontal lobe and hippocampus.

Intoxication with trimethyltin (TMT) leads to profound behavioral and cognitive deficits in both humans [30] and experimental animals [7, 10]. TMT is known to be widely used as plastic stabilizers, wood preservatives, anti-corrosion coatings, pesticides, and kill snails, as well as applied by chemical disinfector and sterilization. So it is clinically meaningful that humans as well as animals can be exposed to it in living environment. In rats, TMT induced the degeneration of the pyramidal neurons in the hippocampus and the cortical areas (pyriform cortex, entorhinal cortex, and subiculum) connected to the hippocampus, but there was also neuronal loss in the association areas [6, 11, 12, 14, 15]. Furthermore, behavioral studies have shown increased disruption of the memory, and learning deficits of TMT-intoxicated rats [20]. TMT intoxication impairs the acquisition of water maze performance [31]. The memory impairment produced by TMT in the current study is consistent with the previous reports of learning impairments [14, 31–33]. During the training trial in a step-through inhibitory avoidance task after shock, there were no differences among the groups. Treatment of Squid-PS seems to be more effective in reversing the memory impairment of late phase, rather than early phase, suggesting that it may facilitate process of memory consolidation. It has been previously reported that PS has profound curative effects on improving the memory and cognitive function of an Alzheimer's disease-like animal model [22, 34]. Zanotti et al. also showed that the treatment of PS in a scopolamine-induced animal model enhanced the learning and memory abilities of the rats [34]. But there was no study using Squid-PS in TMT intoxication model yet.

The effects of Squid-PS on the central acetylcholine system were also examined by performing immunohistochemistry of the hippocampal neurons. The degeneration of the cholinergic innervation from the basal forebrain to the hippocampal formation in the temporal lobe is thought to be one of the factors determining the progression of memory decay, both during normal aging and AD [35]. The best available marker for cholinergic neurons in the basal forebrain is ChAT activity. ChAT synthesizes the neurotransmitter acetylcholine in the basal forebrain, cortex, hippocampus, and amygdala. A significant reduction in ChAT activity in the postmortem brains of demented patients has been reported. In addition, there was a 20–50% decrease in ChAT activity in the hippocampus of the TMT-induced rats in this current study. However, the present results show that Squid-PS exerts beneficial effects on cholinergic neurotransmission in the brain by increasing the hippocampus ChAT activities. Also, acetylcholine esterase (AchE) is an enzyme which breaks down acetylcholine and is a well-known target and biomarker for memory dysfunction or dementia [20]. In AchE histochemistry, the Squid-PS group showed higher AchE reactivity in both hippocampal CA1 and CA3. These results are consistent with previous reports showing that the cholinergic neurons in the brain are involved in learning and memory in humans and animals [36, 37]. In particular, the hippocampal cholinergic neurons are involved in the formation and maintenance of short-term working memory or retention and retrieval processes in long-term reference memory [16, 18, 38–41]. Based on a previous study, this result suggests that the Squid-PS treatment can promote the memory function.

The name “PET” comes from Positron Emission Tomography. It is a new scanning technique in medical research. Small animal PET experiments can be performed using a variety of dedicated small animal scanners (ATLAS, RatPET, microPET). It is a functional imaging modality at molecular level and provides valuable insights into biochemical, physiological, pathological or pharmacological process in vivo. Recent research efforts find its application in a wide area, ranging from basic insights into the normal physiology and disease processes to drug and radiotracer development and gene therapy. The present study showed that the PET analysis, the cerebral glucose metabolism of the Squid-PS datasets was significantly increased in the hippocampus and frontal lobe as compared to the control. An obvious limitation of our study is that the spatial resolution of the present micro-PET system is not high enough to permit more specific analysis of the activity changes within certain brain structures. Nevertheless, there have been several studies that have investigated the brain activity changes in small animals using micro-PET technology [2, 20]. Thus, an important point of our study is that in spite of the limited spatial resolution of the micro-PET system, we were able to detect the TMT-induced focal brain changes.

In summary, treatment with Squid-PS attenuated the TMT-induced learning and memory deficits in the passive avoidance test, and Squid-PS treatment had a protective effect against a TMTinduced decrease of the cholinergic neurons and neural activation. Thus, Squid-PS is a good candidate of neuroprotective agent for treatment of Alzheimer's disease. Further studies that will examine the effects of Squid-PS activation on additional behavioral test will help to elucidate whether increasing the central cholinergic signaling may also improve other types of memory.

## Figures and Tables

**Table 1:** It shows the results of voxelwise comparison between Squid-PS and control datasets. In SPM analysis, the cerebral glucose metabolism of Squid-PS datasets was increased significantly in the hippocampus and frontal lobe compared to control.

Brain area	Coordinates (*x*, *y*, *z*)	*Z* value
Frontal lobe	(−2.4, 2.7, 8.1)	2.14
Hippocampus (Right)	(7.5, 2.1, 2.4)	2.85
Hippocampus (Left)	(−6.5, 2.1, 2.5)	2.13
